# Enhancing Approaches to Inverted Papilloma Through Computed Tomography-Based Hyperostosis Analysis

**DOI:** 10.7759/cureus.78586

**Published:** 2025-02-05

**Authors:** Teru Ebihara, Kazuhiro Omura, Soichiro Fukuzato, Nei Fukasawa, Nobuyoshi Otori

**Affiliations:** 1 Department of Otorhinolaryngology, The Jikei University School of Medicine, Tokyo, JPN; 2 Department of Pathology, The Jikei University School of Medicine, Tokyo, JPN; 3 Department of Otorhinolaryngology, The Jikei University Hospital, Tokyo, JPN

**Keywords:** computed tomography, hyperostosis, inverted papilloma, maxillary sinus, skull base

## Abstract

Objectives: The postoperative recurrence rate of sinonasal inverted papilloma (IP) is high, and the residual tumor at the attachment site (the pedicle of the tumor) is considered the main cause of recurrence. Therefore, a surgical approach tailored to the tumor attachment is crucial. Localized hyperostosis is an imaging characteristic of the attachment observed on computed tomography (CT). This study aimed to determine the tendency of hyperostosis at the IP attachment location according to the detailed anatomical site and to improve the prediction accuracy of the preoperative attachment site.

Materials and methods: This single-center retrospective cohort study was conducted at the Jikei University Hospital from April 2018 to March 2024, targeting patients diagnosed with IP. The attachment distribution and details of the hyperostosis at the attachment on CT were investigated.

Results: Among the 127 included patients, the attachments were identified in the nasal septum (n=3), ethmoid sinus (n=51), maxillary sinus (n=59), frontal sinus (n=8), sphenoid sinus (n=5), and multiple or broad attachments (n=1). Hyperostosis was observed in all cases of the skull base, uncinate process, infraorbital wall, posterior wall, floor of the maxillary sinus, and floor of the sphenoid sinus. In contrast, non-hyperostosis was observed in all cases of the nasal septum, supraorbital cell, anterior wall of the maxillary sinus, and posterior wall of the sphenoid sinus.

Conclusions: This study revealed significant hyperostosis in the posterior wall of the maxillary sinus, infraorbital wall, skull base, uncinate process, and floor of the sphenoid sinus, whereas the infraorbital wall, posterior wall, floor of the maxillary sinus, skull base, uncinate process, and floor of the sphenoid sinus exhibited significant bone hyperostosis. These findings can lead to improved accuracy of preoperative attachment prediction using CT, appropriate surgical approach selection, and better explanations for patients, especially in the maxillary sinus and skull base IP.

## Introduction

Inverted papilloma (IP) is the most common benign sinonasal tumor, with an incidence of 0.2-0.6 cases per 100,000 people, accounting for 0.5-4% of all nasal cavity tumors [1]. Surgical resection is the main treatment strategy, although it has a high postoperative recurrence rate, ranging from 20% to 39% [2,3]. An IP invades the underlying tissues, including bone [4], and procedures beyond mucosal peeling and macroscopic tumor removal are often required. Residual tumor at the attachment site is considered the main cause of recurrence [5,6]. Therefore, a surgical approach tailored to the tumor attachment, known as an “attachment-oriented operation,” is crucial. Regarding specific resection methods, groups undergoing mucosal removal and bone drilling followed by cauterization have a lower recurrence rate than those undergoing mucosal removal only or with bone drilling. These results highlight the importance of thoroughly treating attachment sites [7,8].

The IP imaging characteristics necessary for attachment identification include localized bone hyperostosis, bone prominence, bone thinning, and intratumoral calcification on computed tomography (CT) and a convoluted cerebriform pattern on magnetic resonance imaging (MRI). Among these, localized bone hyperostosis has been repeatedly reported as an imaging predictor for IP attachment [9]. Regarding attachment location distribution, a 2021 systematic review examined 585 IP cases and classified attachment locations into eight categories: lateral nasal wall (13.2%), maxillary sinus (50.9%), ethmoid sinus (9.4%), frontal sinus (5.5%), sphenoid sinus (11.5%), skull base (8.7%), nasal septum (0.7%), and inferior turbinate (0.2%) [10]. A 2023 report on 139 IP cases also classified attachment locations into nine categories [11]. However, there have been few detailed reports on attachment distribution and almost no reports on finer distributions within each sinus, like the maxillary sinus's anterior, posterior, and lateral walls. Previous studies have reported that CT scans have a high accuracy in predicting IP attachment (89-100%) [12-15]. However, these data are limited to studies of 24-50 cases, and accurate prediction of tumor attachment in clinical practice is difficult and questionable. One study with a larger sample size of 143 cases reported an accuracy of 50% [9]. The problem with IP attachment prediction is that limited research has been conducted to classify the finer levels within the paranasal sinuses. More detailed investigations are required to identify the preoperative attachment site accurately. When the preoperative assumed site differs from the actual tumor attachment site, it may lead to postoperative recurrence due to residual attachment, the addition of unexpected surgical approaches not explained beforehand, unexpected bleeding, and prolonged surgery time.

This study aimed to identify detailed trends of bone hyperostosis at IP attachment in CT examinations by anatomical sites and improve the accuracy of preoperative attachment predictions. Utilizing our advantage of accurately identifying detailed attachment locations intraoperatively, we retrospectively evaluated the distribution of attachment sites and bone hyperostosis presence by examining preoperative CT images.

## Materials and methods

We conducted a single-center retrospective cohort study at the Jikei University Hospital from April 2018 to March 2024, targeting patients diagnosed with IP. The inclusion criteria comprised patients who (1) had histologically confirmed IP based on postoperative pathological examination, (2) had preoperative CT scan images (axial, coronal, and sagittal views with a slice thickness of 1 mm), (3) had surgical reports or medical records documenting tumor attachment, and (4) underwent surgery performed by three surgeons using a standardized surgical approach. Preoperatively diagnosed IP patients were 139 cases, and this study included 127 cases of IP treated at our hospital, excluding 12 cases postoperatively diagnosed as malignant.

**Figure 1:**
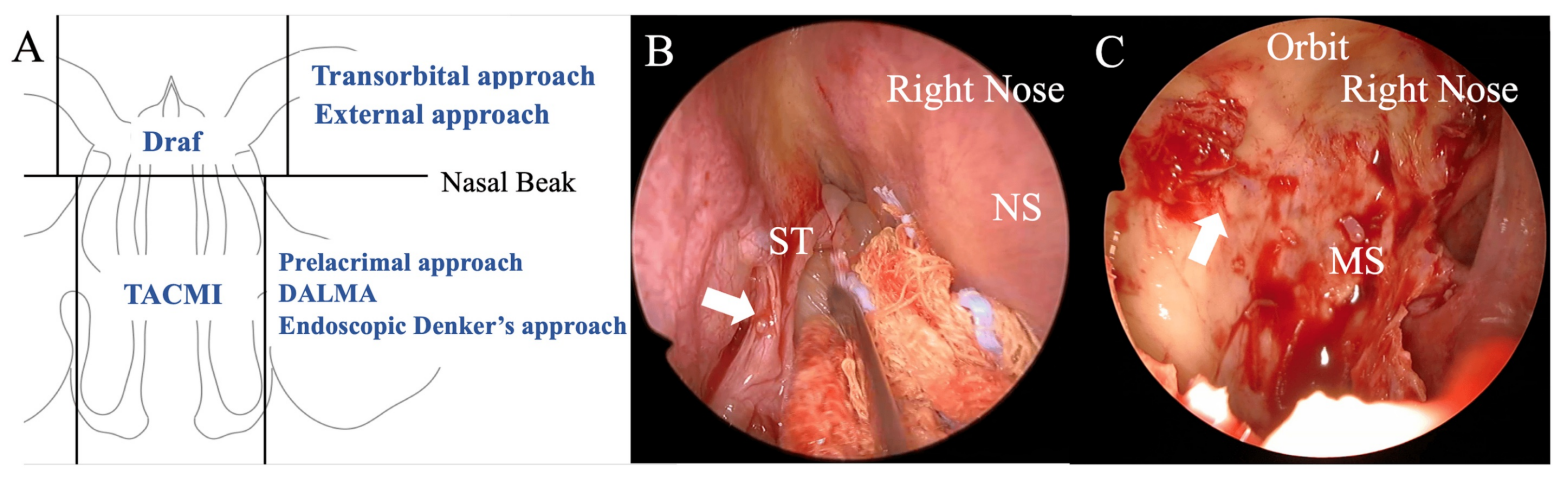
Surgical approaches for sinonasal IP at the Jikei University Hospital and endoscopic imaging findings of IP attachment (A) Surgical approaches for IP at the Jikei University Hospital. The Draf procedure is performed in cases that extend into the frontal sinus. The prelacrimal approach is used for cases extending into the maxillary sinus. In cases where the base is located lateral to the second branch of the trigeminal nerve, DALMA is employed. In cases where the tumor occupies the nasal cavity and touches the nasal septum, TACMI is performed, which involves temporal incision and reconstruction of the nasal septum. (B) Right nasal cavity, 0-degree endoscope imaging findings of IP attachment. IP with the superior turbinate of the right nasal cavity as the attachment (indicated by the arrowhead). (C) Right nasal cavity, 0° endoscope imaging findings of IP attachment after resection of the right maxillary sinus papilloma. A thickened attachment bone (arrowhead) is observed on the lateral wall of the right maxillary sinus. IP: inverted papilloma, ST: superior turbinate, NS: nasal septum, MS: maxillary sinus, DALMA: direct approach to the anterior and lateral part of the maxillary sinus with an endoscope, TACMI: transseptal access and crossing multiple incisions Image Credits: Teru Ebihara

It is difficult to predict the attachment site preoperatively, and it is also difficult to identify the attachment site intraoperatively. Due to the limitations of working space, the common procedure is to incise the tumor and reduce its volume before identifying the attachment. However, when the tumor is incised before identifying the attachment, bleeding often occurs. Although it is possible to identify the attachment at the paranasal sinus level roughly, it is difficult to identify the finer attachment site. Considering the possibility of malignant IP transformation at our institution, we perform surgery using an approach that allows attachment identification before tumor processing whenever possible (Figure 1A). This surgical strategy allows for accurate tumor attachment identification with minimal bleeding in a wide working space (Figure 1B-1C) [16,17].

**Figure 2:**
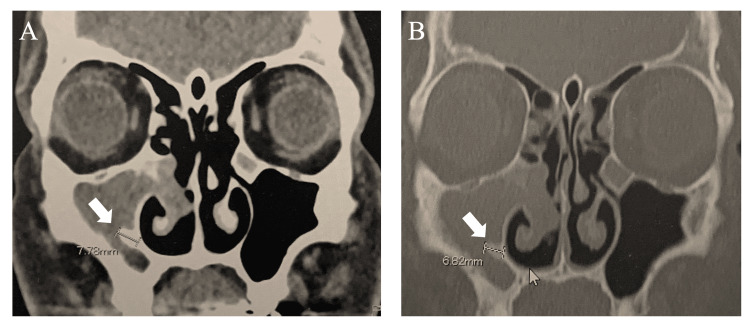
Bone hyperostosis at the IP attachment site on CT (A) Soft tissue density settings. (B) Bone window settings. Bone hyperostosis is observed in the medial wall of the maxillary sinus (indicated by the white arrow). CT: computed tomography, IP: inverted papilloma

The collected data included age, sex, primary or revision surgery, presence of localized bone hyperostosis on CT, tumor attachment identified intraoperatively, and presence or absence of recurrence. Bone hyperostosis was defined as a localized thickening of the nasal/sinus wall on CT compared to the contralateral side [10]. Two senior otolaryngologists independently assessed the presence of localized bone thickening on CT using both bone and soft tissue settings, as in routine clinical practice, and only cases that both the otolaryngologists assessed to have bone thickening were defined as having bone thickening (Figure 2). The postoperative follow-up protocol included outpatient visits every three months for nasal endoscopy and annual MRI scans; the follow-up period duration was from the surgery date until June 30, 2024.

This research was approved by the Ethics Committee of the Jikei University School of Medicine (approval number: 31-244, serial number: 9743).

## Results

Of the 127 patients included in this study, 93 were males (73%) and 34 were females (27%); their median age was 57 years (Table 1). Preoperative CT scans showed bone hyperostosis at the attachment site in 81 patients (64%), whereas 46 patients (36%) did not exhibit bone hyperostosis. A total of 110 primary (87%) and 17 revision (13%) surgeries were performed. En-bloc resection was achieved in 81 patients (64%).

**Table 1:** Patient characteristics stratified by hyperostosis

	Total, n	Hyperostosis, n (%)	No hyperostosis, n (%)
Patients	127	81 (64)	46 (36)
Age (mean)	57	56	59
Sex			
Males	93	63 (68)	30 (32)
Females	34	18 (53)	16 (47)
Follow-up period (month)	32	34	30
Surgery			
Primary	110	71 (65)	39 (35)
Revision	17	10 (59)	7 (41)
En-bloc resection			
Yes	81	51 (63)	30 (37)
No	46	30 (65)	16 (35)
Recurrence			
Yes	0	0	0
No	127	81 (64)	46 (36)
Site of origin			
Nasal septum	3	0	3 (100)
Ethmoid	51	33 (65)	18 (35)
Maxillary	59	39 (66)	20 (34)
Frontal	8	5 (63)	3 (38)
Sphenoid	5	4 (80)	1 (20)
Multiple/broad	1	0	1 (100)

The average follow-up period was 32 months, and none of the 127 patients experienced recurrence. The attachment site distributions were three in the nasal septum (0 cases with bone hyperostosis, all of the perpendicular plate of the ethmoid bone), 51 in the ethmoid sinus (33 with bone hyperostosis, 65%), 59 in the maxillary sinus (39 cases with bone hyperostosis, 66%), eight in the frontal sinus (five cases with bone hyperostosis, 63%), five in the sphenoid sinus (four cases with bone hyperostosis, 80%), and one with multiple or broad bases (0 cases with bone hyperostosis). The attachment was categorized into six regions: nasal septum, ethmoid sinus, maxillary sinus, frontal sinus, sphenoid sinus, and multiple or broad bases.

**Figure 3:**
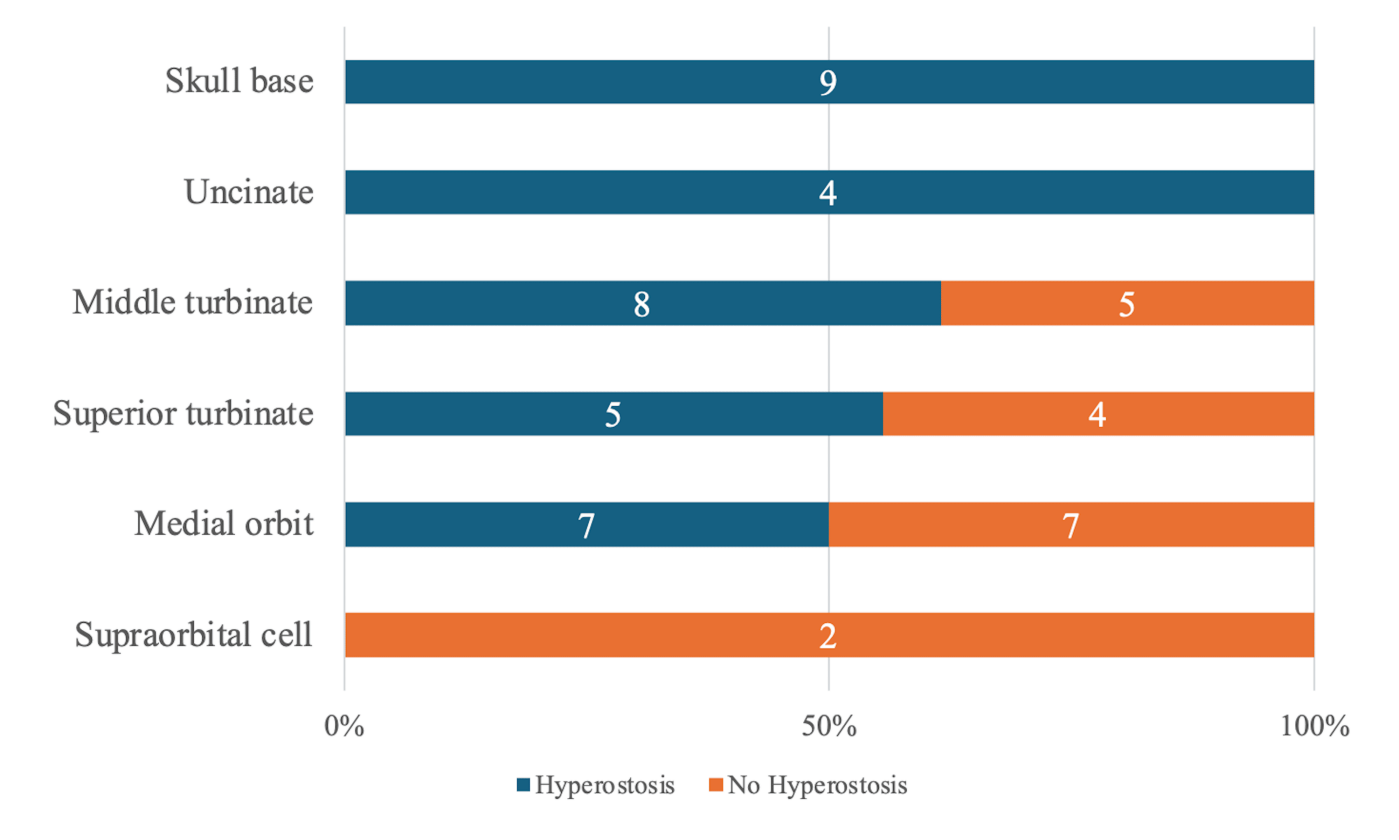
Attachment distribution stratified by ethmoid sinus hyperostosis Blue bars indicate hyperostosis, and orange bars indicate no hyperostosis. The number of cases of hyperostosis and no hyperostosis is labeled in the bar, with the total as 100%. The ethmoid sinus was classified into six categories: skull base (nine cases; nine with bone hyperostosis), uncinate process (four cases; four with bone hyperostosis), middle turbinate (13 cases; eight with bone hyperostosis), superior turbinate (nine cases; five with bone hyperostosis), medial orbital wall (14 cases; seven with bone hyperostosis), and supraorbital cells (two cases; 0 with bone hyperostosis).

A more detailed investigation and evaluation of each sinus were conducted. The ethmoid sinus was classified into six categories: middle turbinate (13 cases; eight with bone hyperostosis), superior turbinate (nine cases; five with bone hyperostosis), skull base (nine cases; nine with bone hyperostosis), uncinate process (four cases; four with bone hyperostosis), medial orbital wall (14 cases; seven with bone hyperostosis), and supraorbital cells (two cases; 0 with bone hyperostosis) (Figure 3).

**Figure 4:**
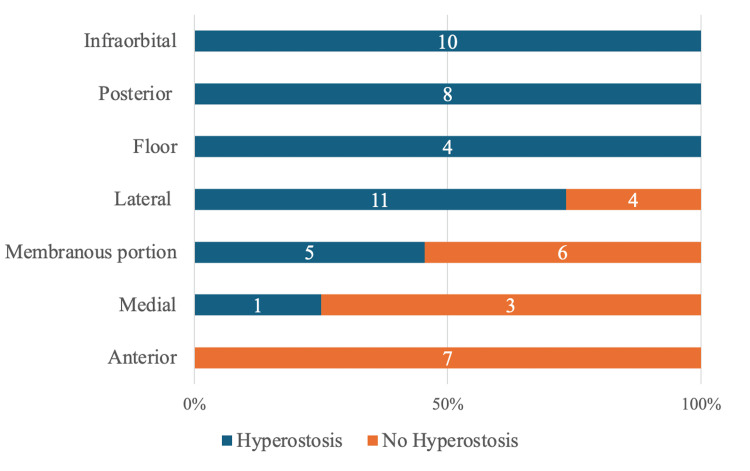
Attachment distribution stratified by maxillary sinus hyperostosis Blue bars indicate hyperostosis, and orange bars indicate no hyperostosis. The number of cases of hyperostosis and no hyperostosis is labeled in the bar, with the total as 100%. The maxillary sinus was classified into seven categories: infraorbital (10 cases; 10 with bone hyperostosis), posterior wall (eight cases; eight with bone hyperostosis), floor (four cases; four with bone hyperostosis), lateral wall (15 cases; 11 with bone hyperostosis), membranous portion (11 cases; five with bone hyperostosis), medial wall (four cases; one with bone hyperostosis), and anterior wall (seven cases; 0 with bone hyperostosis).

The maxillary sinus was classified into seven categories: anterior wall (seven cases; 0 with bone hyperostosis), posterior wall (eight cases; eight with bone hyperostosis), floor (four cases; four with bone hyperostosis), lateral wall (15 cases; 11 with bone hyperostosis), medial wall (four cases; one with bone hyperostosis), orbital floor (10 cases; 10 with bone hyperostosis), and membranous portion (11 cases; five with bone hyperostosis) (Figure 4).

**Figure 5:**
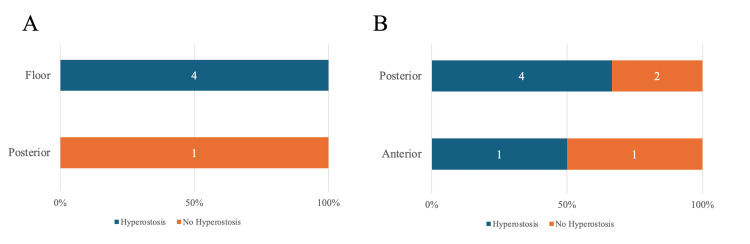
Attachment distribution stratified by sphenoid and frontal sinus hyperostosis Blue bars indicate hyperostosis, and orange bars indicate no hyperostosis. The number of cases of hyperostosis and no hyperostosis is labeled in the bar, with the total as 100%. (A) Attachment distribution stratified by sphenoid sinus hyperostosis. The sphenoid sinus was classified into two categories: floor (four cases; four with bone hyperostosis) and posterior wall (one case; 0 with bone hyperostosis). (B) Attachment distribution stratified by frontal sinus hyperostosis. The frontal sinus was classified into two categories: posterior (six cases; four with bone hyperostosis) wall and anterior wall (two cases; one with bone hyperostosis).

The sphenoid sinus was classified into two categories: posterior wall (one case; 0 with bone hyperostosis) and floor (four cases; four with bone hyperostosis) (Figure 5A). The frontal sinus was classified into two categories: anterior (two cases; one with bone hyperostosis) and posterior (six cases; four with bone hyperostosis) walls, and no significant differences in bone hyperostosis were observed (Figure 5B).

## Discussion

The present study showed no recurrences in the 127 patients diagnosed with IP postoperatively (average follow-up period of 32 months). Previous studies have reported postoperative recurrence rates of 20% and 30% for IP; thus, the surgical outcomes at our institution can be considered relatively favorable [2,3]. Regarding control of benign tumor IP without malignant intermingling, we believe there is no significant difference in outcome between partial resection and en bloc resection. One factor contributing to the good surgical outcomes was our surgical approach, which aims to identify the attachment without incising the tumor as much as possible. This approach allows for a wide working space, manipulating the tumor and exposing the attachment without an incision. Specific methods for ensuring sufficient working space include the Draf procedure for cases extending into the frontal sinus, the prelacrimal approach for cases extending into the maxillary sinus, the direct approach to the anterior and lateral part of the maxillary sinus with an endoscope (DALMA) [16] for cases with an attachment lateral to the second branch of the trigeminal nerve (V2), and transseptal access and crossing multiple incisions [17] for cases occupying the nasal cavity and touching the nasal septum. Even if the final approach involves a tumor incision, identifying the attachment before resection allows for more precise attachment-oriented surgery [18,19]. In the examined data, the overall bone hyperostosis rate was 63.8%, with 64.5% in primary surgeries and 58.8% in revision surgeries, aligning with previous reports [10]. The base bone hyperostosis rates by sinus were 0% in the nasal septum, 66% in the maxillary sinus, 65% in the ethmoid sinus, 63% in the frontal sinus, 80% in the sphenoid sinus, and 0% in cases with multiple or broad bases. Compared with previous data, the overall hyperostosis rate was similar; however, the proportions within each category varied significantly [5].

The analysis revealed that the infraorbital wall, posterior wall, floor of the maxillary sinus, skull base, uncinate process, and sphenoid sinus floor were anatomical sites where the attachment bone was more likely to thicken. In contrast, the nasal septum, anterior wall of the maxillary sinus, supraorbital cell, and posterior wall of the sphenoid sinus were less likely to thicken.

Among the IPs, those originating from the maxillary sinus have poor treatment outcomes, with recurrence rates ranging from 16% to 30% due to the difficulty of complete resection in areas with uneven wall shapes, such as the alveolar and zygomatic recesses [20,21]. The lateral and anterior walls of the maxillary sinus are also difficult to resect. For IPs with attachments to the lateral or anterior walls of the maxillary sinus, the prelacrimal approach alone may not suffice, and combined procedures, such as the Caldwell-Luc operation or DALMA, may be necessary. Our study indicated that, in cases of maxillary sinus IP in which preoperative CT imaging shows no bone hyperostosis, there was a significantly higher likelihood that the anterior wall of the maxillary sinus was attached. This allows for a better explanation for the patient and planning for a combined surgical approach involving the lacrimal approach, Caldwell-Luc operation, or DALMA. Our study also found that bone hyperostosis in the infraorbital and posterior walls of the maxillary sinus were significant indicators for attachment prediction. Care must be taken for lesions on the infraorbital wall to avoid injury to V2 or the orbit during dissection or bone drilling. Lesions at the attachment of the posterior wall of the maxillary sinus often receive blood supplied from the maxillary artery and its branches; therefore, arterial bleeding should be carefully monitored during bone drilling. These findings suggest that IPs on the infraorbital wall and posterior wall of the maxillary sinus can be predicted with higher accuracy, allowing for preoperative patient counseling regarding the risks of V2 or orbital injury, the risk of bleeding from the maxillary artery or its branches, and potential options for preoperative angiography and embolization of the feeding vessels. The present study data contribute to improving preoperative attachment prediction and appropriate surgical approach selection for challenging maxillary sinus IPs and serve as useful information for patient counseling.

Additionally, the present study found that the skull base was a site where the attachment bone was more likely to thicken. Data on IPs originating from the skull base are limited, and these cases are among the most challenging. Treatment involves peeling off the tumor along with the mucosa and performing bone drilling and cauterization at the attachment to reduce the recurrence rate [22]. However, skull base drilling is an advanced technique; the attachment at the skull base is often not identified preoperatively and only discovered intraoperatively. In a review of 86 cases, the skull base had the lowest detection sensitivity by preoperative CT scan (17%). The reason for this was considered to be concurrent opacification and osteitic changes of adjacent sinuses, such as the ethmoid sinuses for the four lesions attached to the lateral lamella and fovea ethmoidalis and the sphenoid sinus for the lesions originating at the planum sphenoidale and temporal fossa superior to the lateral sphenoid recess [23]. The results of the present study indicated that bone hyperostosis in the skull base on CT was a predictor for attachment identification and was very useful for preoperative planning of skull base bone drilling and flap coverage, as well as for patient counseling regarding the possibility of cerebrospinal fluid leakage and the need for fat or fascia lata harvesting for skull base reconstruction.

The major limitation of this study is that the small sample size of each of the detailed attachment sites examined did not allow for a statistical study; we require further investigation and case accumulation. Moreover, in the revision cases, we considered bone hyperostosis when there was obvious localized thickening of the nasal bone. However, one limitation is that this is a single-center study at our institution, not a multicenter one. In addition, in the case of the attachment of the nasal septum, its cartilaginous component may influence the results and is a limitation.

## Conclusions

We reported a detailed analysis of the attachment distribution of sinonasal IP and its propensity for hyperostosis. The nasal septum, supraorbital cell, anterior wall of the maxillary sinus, and posterior wall of the sphenoid sinus showed non-hyperostosis. In contrast, the infraorbital wall, posterior wall, floor of the maxillary sinus, skull base, uncinate process, and floor of the sphenoid sinus exhibited significant bone hyperostosis. These results may improve the accuracy of attachment prediction using preoperative CT, selecting the appropriate surgical approach, and enhancing patient counseling, especially in the maxillary sinus and skull base IP.

## References

[1] Lund VJ, Stammberger H, Nicolai P (2010). European position paper on endoscopic management of tumours of the nose, paranasal sinuses and skull base. Rhinol Suppl.

[2] Miao S, Cheng Y, Li Y, Chen X, Chen F, Zha D, Xue T (2024). Prediction of recurrence-free survival and risk factors of sinonasal inverted papilloma after surgery by machine learning models. Eur J Med Res.

[3] He X, Wang Y (2021). Clinical characteristics of sinonasal inverted papilloma associated with recurrence and malignant transformation. Auris Nasus Larynx.

[4] Chiu AG, Jackman AH, Antunes MB, Feldman MD, Palmer JN (2006). Radiographic and histologic analysis of the bone underlying inverted papillomas. Laryngoscope.

[5] Kim JS, Kwon SH (2017). Recurrence of sinonasal inverted papilloma following surgical approach: a meta-analysis. Laryngoscope.

[6] Tong CC, Patel NN, Maina IW (2019). Inverted papilloma with multifocal attachment is associated with increased recurrence. Int Forum Allergy Rhinol.

[7] Tomenzoli D, Castelnuovo P, Pagella F (2004). Different endoscopic surgical strategies in the management of inverted papilloma of the sinonasal tract: experience with 47 patients. Laryngoscope.

[8] Trent MS, Goshtasbi K, Hui L, Stuyt JA, Adappa ND, Palmer JN, Kuan EC (2022). A systematic review of definitive treatment for inverted papilloma attachment site and associations with recurrence. Otolaryngol Head Neck Surg.

[9] Fang G, Lou H, Yu W (2016). Prediction of the originating site of sinonasal inverted papilloma by preoperative magnetic resonance imaging and computed tomography. Int Forum Allergy Rhinol.

[10] Glikson E, Dragonetti A, Soudry E (2021). Can computed tomography findings predict the recurrence of sinonasal inverted papilloma?. Otolaryngol Head Neck Surg.

[11] Yeom S, Lee DH, Lim SC (2023). Clinical outcomes of sinonasal inverted papilloma: a retrospective analysis of 139 cases. J Laryngol Otol.

[12] Bhalla RK, Wright ED (2009). Predicting the site of attachment of sinonasal inverted papilloma. Rhinology.

[13] Lee DK, Chung SK, Dhong HJ, Kim HY, Kim HJ, Bok KH (2007). Focal hyperostosis on CT of sinonasal inverted papilloma as a predictor of tumor origin. AJNR Am J Neuroradiol.

[14] Sham CL, King AD, van Hasselt A, Tong MC (2008). The roles and limitations of computed tomography in the preoperative assessment of sinonasal inverted papillomas. Am J Rhinol.

[15] Yousuf K, Wright ED (2007). Site of attachment of inverted papilloma predicted by CT findings of osteitis. Am J Rhinol.

[16] Omura K, Nomura K, Aoki S, Otori N, Tanaka Y (2019). Direct approach to the anterior and lateral part of the maxillary sinus with an endoscope. Auris Nasus Larynx.

[17] Omura K, Nomura K, Aoki S, Hosokawa Y, Tanaka Y, Otori N, Kojima H (2020). Resection of inverted papilloma in nasal cavity with transseptal access and crossing multiple incisions minimizes bleeding and reveals the tumor pedicle. Auris Nasus Larynx.

[18] Attlmayr B, Derbyshire SG, Kasbekar AV, Swift AC (2017). Management of inverted papilloma: review. J Laryngol Otol.

[19] Wang Y, An Y, Zhao C, Dong R, Cheng F (2020). Attachment-oriented endoscopic treatment of inverted papilloma involving the frontal sinus/recess. J Craniofac Surg.

[20] Vinciguerra A, Mattavelli D, Turri-Zanoni M (2023). Validation of modular endoscopic medial maxillectomies for inverted papilloma of the maxillary sinus. Rhinology.

[21] Healy DY Jr, Chhabra N, Metson R, Holbrook EH, Gray ST (2016). Surgical risk factors for recurrence of inverted papilloma. Laryngoscope.

[22] Kuan EC, Wang EW, Adappa ND (2024). International consensus statement on allergy and rhinology: sinonasal tumors. Int Forum Allergy Rhinol.

[23] Lee JJ, Orlowski HL, Schneider JS (2021). Computed tomography as a predictor of sinonasal inverted papilloma origin, skull base involvement, and stage. J Neurol Surg B Skull Base.

